# Exploration of Novel Pathways Underlying Irreversible Electroporation Induced Anti-Tumor Immunity in Pancreatic Cancer

**DOI:** 10.3389/fonc.2022.853779

**Published:** 2022-03-18

**Authors:** Khan Mohammad Imran, Margaret A. Nagai-Singer, Rebecca M. Brock, Nastaran Alinezhadbalalami, Rafael V. Davalos, Irving Coy Allen

**Affiliations:** ^1^ Graduate Program in Translational Biology, Medicine and Health, Virginia Tech, Roanoke, VA, United States; ^2^ Department of Biomedical Sciences and Pathobiology, Virginia Tech, Blacksburg, VA, United States; ^3^ Institute for Critical Technology and Applied Sciences, Virginia Tech, Blacksburg, VA, United States; ^4^ Department of Biomedical Engineering and Mechanics, Virginia Tech, Blacksburg, VA, United States; ^5^ Department of Mechanical Engineering, Virginia Tech, Blacksburg, VA, United States

**Keywords:** pancreatic cancer, irreversible electroporation, anti-tumor immunity, immunomodulatory pathways, IFNγ-PD-L1 axis

## Abstract

Advancements in medical sciences and technologies have significantly improved the survival of many cancers; however, pancreatic cancer remains a deadly diagnosis. This malignancy is often diagnosed late in the disease when metastases have already occurred. Additionally, the location of the pancreas near vital organs limits surgical candidacy, the tumor’s immunosuppressive environment limits immunotherapy success, and it is highly resistant to radiation and chemotherapy. Hence, clinicians and patients alike need a treatment paradigm that reduces primary tumor burden, activates systemic anti-tumor immunity, and reverses the local immunosuppressive microenvironment to eventually clear distant metastases. Irreversible electroporation (IRE), a novel non-thermal tumor ablation technique, applies high‐voltage ultra-short pulses to permeabilize targeted cell membranes and induce cell death. Progression with IRE technology and an array of research studies have shown that beyond tumor debulking, IRE can induce anti-tumor immune responses possibly through tumor neo-antigen release. However, the success of IRE treatment (i.e. full ablation and tumor recurrence) is variable. We believe that IRE treatment induces IFNγ expression, which then modulates immune checkpoint molecules and thus leads to tumor recurrence. This indicates a co-therapeutic use of IRE and immune checkpoint inhibitors as a promising treatment for pancreatic cancer patients. Here, we review the well-defined and speculated pathways involved in the immunostimulatory effects of IRE treatment for pancreatic cancer, as well as the regulatory pathways that may negate these anti-tumor responses. By defining these underlying mechanisms, future studies may identify improvements to systemic immune system engagement following local tumor ablation with IRE and beyond.

## Introduction

The advances of cancer treatments have been staggering in the last 50 years. From advancing chemotherapeutics to cytokine and antibody treatments, many cancer patients have seen a significant increase in survival rates. However, some cancers have continued to be relatively unaltered by these new treatments (1–3). Dense tissue in later-stage cancers, as well as several other factors, can limit tumor penetration and response of drugs (4). In the past decade, immunotherapeutic developments, including checkpoint inhibitor monoclonal antibodies (mAbs) and adoptive cellular therapy, have shown promise in treating many cancer patients with advanced-stage tumors (5, 6). However, cancers such as pancreatic adenocarcinoma continue to have severely low survival rates even with new immunotherapeutic options in part due to the immunosuppressive tumor microenvironment that shields the tumors from the immune system’s attempts to identify and target the malignancy (7, 8). Pancreatic cancer has a low incidence rate (3.2% of all new cancer cases) but high death rate (7.9% of all cancer related death), making it the third-highest cause of cancer-related deaths in the United States (9). Additionally, pancreatic cancer is projected to become the second-highest cause of cancer-related deaths by 2030 (10, 11). It is often diagnosed late in the disease progression, which severely limits treatment options. Furthermore, due to close proximity to critical structures, the standard-of-care surgery is only available to 20% of total diagnosed patients.

These limitations have led to a rush of targeted ablation modalities to circumvent the challenges faced by surgeons and oncologists. Many of these modalities use thermal effects that burn or freeze the tumor. However, these technologies can lead to adverse effects and limited application by causing off-target damage to nearby healthy tissues, and heat sink effect making them unsuitable for many cancer patients (12, 13). The use of thermal energy can also denature proteins, which can limit immune signaling by destroying potential damage signals and tumor antigens (14, 15). Non-thermal ablation modalities, such as those utilizing electroporation, greatly limit damage to surrounding healthy tissues (16, 17). Moreover, immunogenic signaling could be preserved, allowing for damage signals and viable antigens to remain intact and elicit an anti-tumor immune response after treatment (18, 19). Preclinical and clinical reports of potential immunological effects to the primary treatment site and even metastatic lesions have led to many recent investigations on the impact of these electroporation-based modalities, such as irreversible electroporation (IRE), high-frequency irreversible electroporation (H-FIRE), electrochemotherapy (ECT), and nanosecond pulse electric fields (nsPEFs) on the tumor microenvironment and the immune system. Indeed, several reports suggested an abscopal-like effect where treatment of the primary tumor by electroporation-based modalities resulted in decreased tumor size and reduced distant metastases (20–22).

A plethora of signaling pathways are involved in the formation and progression of pancreatic cancer. Many of these pathways involve cell death/survival and immune system activation/suppression and have been observed after IRE treatment. Elucidating these pathways, which are poorly understood in the context of IRE ablation of pancreatic cancer, could potentially change its clinical application. Such discoveries could inform well-rounded treatment plans that limit IRE side effects (tumor lysis syndrome, organ damage from inflammation, and developing autoimmune diseases) and work alongside chemotherapies or immunotherapies. It would also provide much-needed information on how IRE impacts the tumor microenvironment and potential improvements to the treatment modality itself. A better understanding of these mechanisms can thus lead to improved patient outcomes and prolonged survival. In this review, we discuss pathways involved in cell death, survival, immune system activation, and immune suppression after IRE treatment.

## IRE Ablation Releases Tumor Antigen

**Figure 1:**
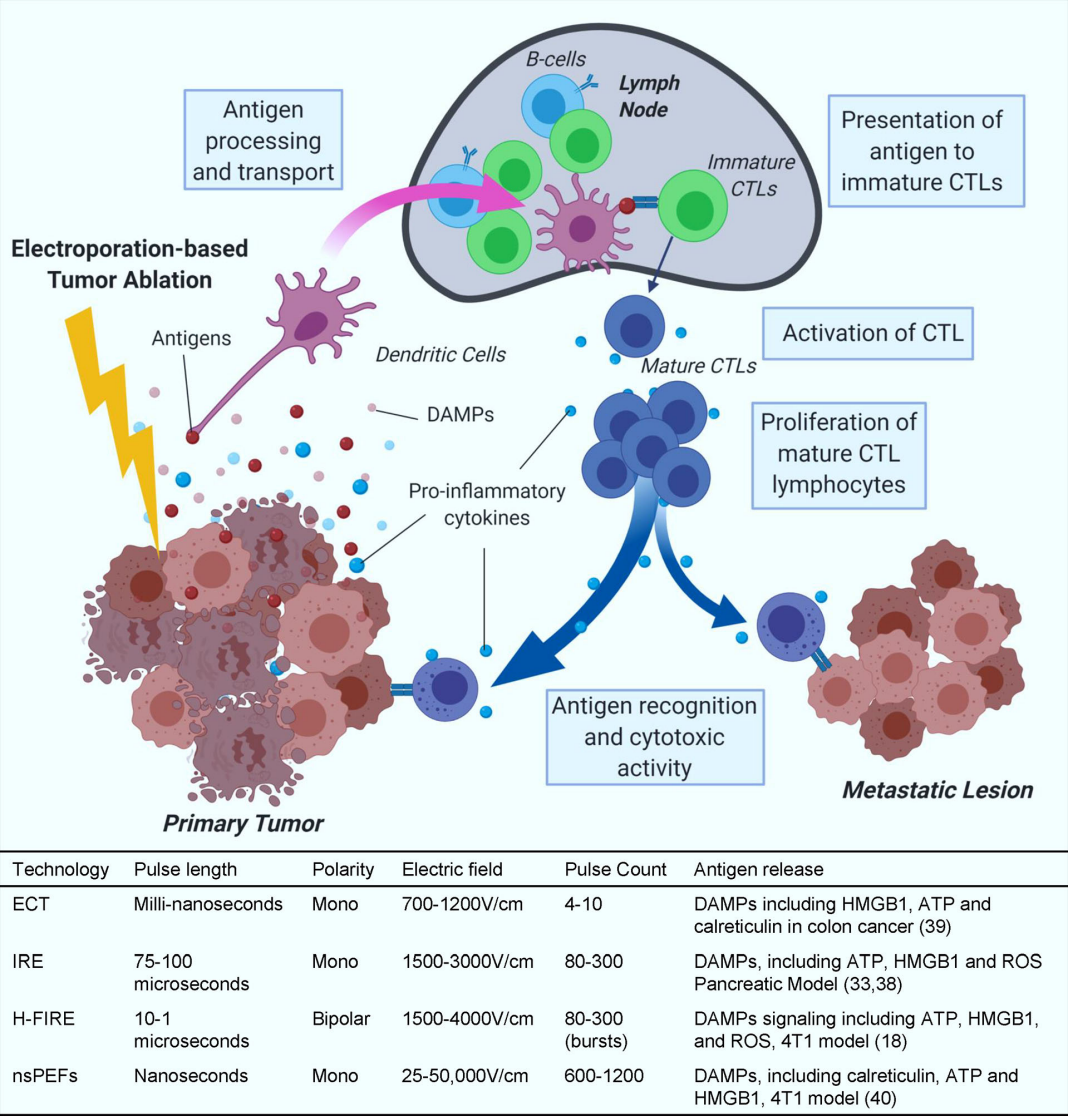
Antigen release and presentation. Collection and presentation of antigens by dendritic cells to cytotoxic T-cells (CTLs) and subsequent CTL activity after IRE treatment. Treatment parameter value ranges are based on commonly reported parameters in literature for *in vivo* preclinical and clinical applications. These ranges are not definite and applications outside of these ranges can occur.

Electroporation-based ablations include many subtypes of ablative strategies, from ECT to nsPEFs (**Figure 1**). These different treatments require adjustments of pulsed electric fields (PEFs) to generate the desired tumor ablation. By adjusting the polarity, duration, electric field strength (V/cm), and number of pulses applied, electroporation can temporarily or permanently permeabilize cell membranes. European Standard Operating Procedure on Electrochemotherapy (ESOPE) multicenter trial has standardized Electrochemotherapy treatment parameters which was first published in 2006 and recently updated (23–25). The ESOPE protocol (8 rectangular pulses, 1000 V/cm, 100 microseconds) specifies the standard pulsing requirements on human patients. In recent years, IRE has been integral to many clinical trials targeting notoriously difficult-to-treat malignancies, including liver and pancreatic cancers (26–29). The significant results of recent clinical trials have propelled IRE into Phase III clinical trials in pancreatic cancer (clinicaltrials.gov, ID: NCT03899636) (30). IRE utilizes microsecond pulsed electric fields. Unlike ECT, IRE increases the number of applied pulses so that so that cells cannot recover from the membrane permeabilization and induce cell death through a disruption in homeostasis (31). IRE is often compared to thermal ablation treatments, but it is normally applied non-thermally and thereby reduces the risk of healthy tissue damage (16) and spares critical structures (29, 32).

IRE treatment induces different types of cell death, such as apoptosis and necrosis in pancreatic cancer cell lines (33). Treating with next-generation IRE, known as H-FIRE, induces apoptosis, necrosis and pyroptosis in liver and breast cancer (18, 34). After IRE treatment, cytoplasmic and antigenic materials are released through the permanent damage of the cell membrane and lead to different types of cell death. These findings were recently summarized by Brock et al. (35). Apoptosis is more controlled and promotes a weak immune response due, in part, to minimal debris and antigen released after programmed phagocytosis (36). In contrast, necrosis involves rapid accidental or mechanical lytic cell death that leads to the release of large amounts of damage-associated molecular patterns (DAMPs). Necrosis-released DAMPs can activate the innate immune system, induce inflammation, and recruit immune cells to the local tumor site. Pyroptosis is a tightly regulated inflammatory form of programmed cell death distinguished from necrosis by the cleavage and activation of Caspase-1 and Caspase-11 (37). During pyroptosis, a substantial quantity of DAMPs, including adenosine triphosphate (ATP), High mobility group box protein 1 (HMGB1), reactive oxygen species (ROS), and different potent proinflammatory cytokines are released, including IL-1β and IL-18 (38). IRE treatment to KrasLSL-G12D-p53LSL-R172H-Pdx-1-Cre (KPC) pancreatic cancer cells produces DAMPs validated by the production of ATP (33). Likewise, H-FIRE treatment has been shown to upregulate several DAMPs such as ATP, HMGB1, and ROS (18). ECT promotes LCs migration from the tumor to draining lymph nodes and pDCs and dDCs recruitment at the site of the lesion (39). Furthermore, ECT and nsPEF have also been shown to release several DAMPs including ATP, HMGB1, and calreticulin in different cancer models (40, 41). Although several studies have shown positive immune response after IRE for pancreatic cancer (22, 38, 42–44), more research needs to be done to determine the immune response after ECT due to different cell death pathways.

It is well established that DAMPs including HMGB1, heat shock protein (HSP), and antigens released *via* immunogenic cell death can activate the immune system against specific cancer cells (45, 46). It is evident that IRE causes immunogenic cell death and helps release tumor antigens which should, in turn, activate systemic anti-tumor immunity against pancreatic cancer and change the tumor microenvironment (**Figure 1**). By improving the microenvironment, the tumor is accessible to anti-tumor leukocytes such as neutrophils, macrophages, natural killer (NK) cells, T helper 1 cells, and CTLs (47–50). Indeed, many cancer patients show an increase in survival outcomes when more activated immune cells and less immune suppressive cells are observed (24, 28, 51–55),. These findings indicate the importance of monitoring and altering the immune system activation in cancer patients to improve survival.

## Released Tumor Antigen by IRE Is Presented by APCs to Activate T Cells

Dendritic cells, macrophages, and B cells act as antigen-presenting cells (APCs) through interactions with T cells to link innate and adaptive immune responses. APCs display tumor antigens on the cell surface through major histocompatibility complexes to control the activation, differentiation, and effector functions of T cells. Electroporation-based ablation modalities do not use thermal energy to induce cell death and are believed to preserve viable tumor antigens that can be presented by APCs to T cells to activate systemic antitumor immunity (**Figure 1**). The lack of thermal energy is thought to preserve optimal antigen structure, size, and confirmation for APCs to present. When APCs encounter tumor antigens, they activate naïve T cells either by moving to nearby lymph nodes or locally in the tissue. Mature and activated T cells proliferate and migrate to the local tumor site and circulate systemically (56).

Immunocompetent mice demonstrate enhanced local and systemic anti-tumor efficacy following electroporation compared to immunodeficient mice, which indicates involvement of the immune system in the reduction of tumor burden by IRE treatment (57). We have recently shown that H-FIRE and IRE treated glioblastoma cells can activate CD8+ cytotoxic T cells (CTL) when cultured with CD4+ helper T cells and antigen-presenting dendritic cells (58). A recent report shows that IRE treatment using a mouse model of orthotopic pancreatic cancer resulted in an increased number of cytotoxic CD8+ T cells and memory T cells in the spleen, lymph nodes, and tumor, and induced an abscopal like effect through the synthesis and secretion of DAMPs (22). A significant increase in the CD8+ T cells and macrophages in the tumor has been noted after IRE and nsPEF treatment (59, 60). Macrophage and pan-T cell infiltration has been found from 6 hours to 14 days after IRE treatment (61, 62). A recent review compiled a timeline to show immune cell types and their upregulation or downregulation in the pancreatic tumor site after IRE treatment from both mouse models and human patients (43).

A murine pancreatic tumor model showed that after IRE treatment, there is evidence for antigen release and an increase in T cells in the lymph nodes and activated T cell infiltration in the local tumor site (22). However, direct evidence that tumor antigens released by IRE treatment then processed by APCs and their migration to the lymph nodes are not established. Furthermore, *in situ* T cell responses in the local tumor after IRE treatment are through antigen-mediated activation is not well demonstrated and requires further investigation.

## T Cell-Mediated Cytokine Expression Pathway

Upon initial contact with tumor antigen, activated CD8+ cytotoxic T cells express a plethora of cytokines based on their subtypes and exposure to polarizing cytokines (63) including interferon gamma (IFNγ), Granzyme B, and Perforin (64–66). In a study with 34 locally advanced pancreatic cancer (LAPC) patients, significant upregulation was detected in the blood for IL-2, IL-6, and IL-10 after IRE treatment (62). In another study with 79 Stage III/IV pancreatic cancer patients, IRE treatment along with allogeneic natural killer cell therapy was tested and demonstrated that IRE alone and IRE with NK cell therapy increases IFNγ expression (67). In a murine model of pancreatic cancer, IRE treatment increased IFNγ expression compared to the sham control procedure (33). In an osteosarcoma rat model, IRE induced IFNγ expression in the serum (68). These findings indicate predominantly pro-inflammatory cytokine expression after electroporation-based tumor ablation treatment, presumably by activated lymphocytes.

**Figure 2:**
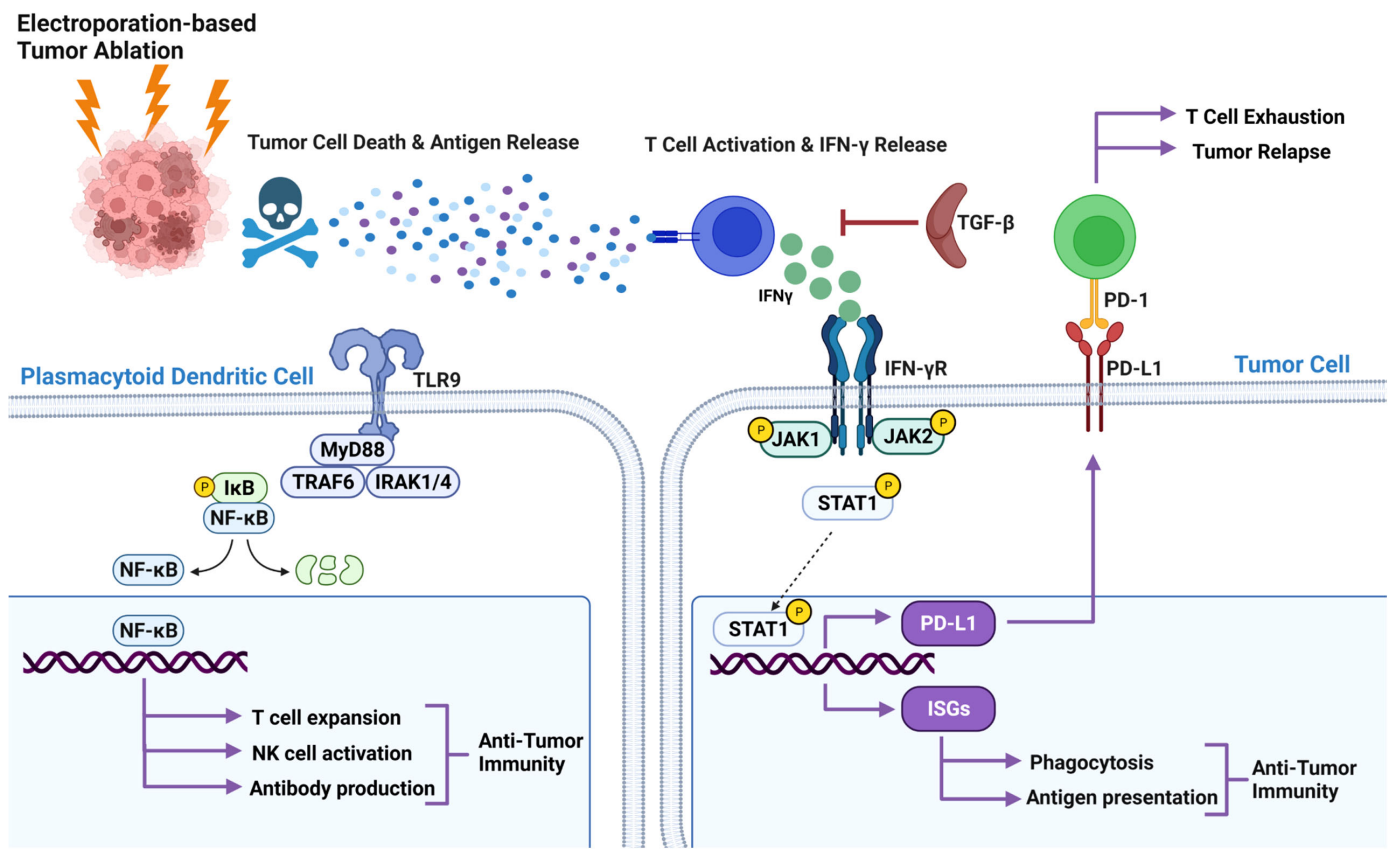
Pathways involved in the IRE treatment of pancreatic cancer. Released antigen after IRE treatment activates CTL and induces IFNγ expression. Binding IFNγ to IFNR recruit and phosphorylate JAK1, and JAK2 activates STAT1 by phosphorylation results in translocation of STAT1 into the nucleus. In the nucleus STAT1 binds to the GAS site and starts the transcription of interferon-stimulated genes (ISGs). ISGs not only encode cytokines and chemokines, but also phagocytic receptors and antigen-presenting molecules. IFNγ also induces PD-L1 expression by tumor cells through the JAK/STAT pathway. TLR9 agonists promote antitumor immunity through NK-κB signaling.

IFNγ is primarily produced by lymphocyte populations, such as NK cells, innate lymphoid cells, T helper 1 (TH1) cells, and CTLs. For different cell types, the signaling can initiate through pattern recognition receptors, T cell receptors, and IFNγR. Stimulation of any of these receptors triggers the recognized Janus kinase (JAK) signal transducer and activator of transcription (STAT) signaling pathway (**Figure 2**). IFNγR ligand binding results in the initiation of JAK1 and JAK2 receptor association and activation followed by phosphorylation and activation of STAT1. Activated STAT1 moves to the nucleus, binds to the GAS site, and starts the transcription of interferon-stimulated genes (ISGs). ISGs not only encode cytokines and chemokines but also phagocytic receptors and antigen-presenting molecules (**Figure 2**). It is apparent that IRE produces tumor antigens that elicit an immune response, specifically *via* APCs that present the tumor antigens to IFNγ-expressing T cells. These activated T cells then kill tumor cells directly or activate alternative killing mechanisms, such as macrophage activation. However, the relationship between antigen presentation, T cell activation, and cytokine expression has not been fully elucidated in the context of IRE. We suggest this interface reveals an exciting co-therapy target, such as enhancing the release and subsequent presentation of a specific antigen and can also inform the timing of co-therapy application.

## IFNγ Mediated PD1/PD-L1 Expression and CTL Inhibition Pathway

Unrestrained immune responses to tumor antigens can cause excessive inflammatory tissue damage and autoimmune diseases, therefore, immune homeostasis is critical for host survival. To maintain this homeostasis, the extent of the immune response is controlled by a balance between co-stimulatory and inhibitory signals of immune checkpoints. These checkpoints are often hijacked by tumor cells to evade the immune system. In a mouse model of pancreatic cancer, IFNγ expression by splenocytes after IRE and PD-L1 expression by tumor cells after IFNγ treatment has been demonstrated (69). An increase in CD4+PD1+ and CD8+PD1+ T cells 2 weeks post-IRE were found in the blood of all 10 LAPC patients (44). Enhanced antitumor efficacy of IRE and anti-PD1 immune checkpoint blockade in a murine orthotopic pancreatic adenocarcinoma (PDAC) model (KRAS model) were found where blocking of CD8a negates the benefit of IRE and blocking of CTLA4 did not enhance the efficacy of IRE. These results denote the importance of retaining CTL’s anti-tumor activity by targeting the right checkpoint molecule (38). IFNγ mediated PD-L1 expression is also regulated by JAK/STAT pathway (**Figure 2**), and emphasis has been put on why anti-PD-L1 immunotherapy might not work if IFNγ mediated PD-L1 is not expressed (70). IFNγ expression after IRE treatment due to T cell activation and PD1 by T cells and PD-L1 by tumor cells have been shown, but it is poorly understood what pathway is being activated and requires further investigation.

Fascinating genetic and biochemical results support crucial roles for JAK1, JAK2, STAT1, and ISGs in facilitating cellular IFNγ responses. The JAK-STAT pathway is also important in IFNγ function, as seen in host defense against pathogens, inflammatory and immune responses, tissue damage, and tumor immunosurveillance. In the context of pancreatic tumors treated with IRE, the IFNγ–JAK–STAT1–ISG pathway, immune functions of ISGs, and feedback inhibition of this pathway pose some key questions to be answered. Many components of this pathway are interlinked. For example, antigens released through IRE treatment activate T cells and produce IFNγ, and IFNγ can induce itself and/or PD-L1 presumably through JAK–STAT1–ISG pathway. IFNγ stimulates its own production through a positive feedback loop and induces PD-L1 through a negative feedback loop, which may be regulated through spacial, temporal and cell type dependent manner. This complex interaction is not yet fully defined in the context of IRE and pancreatic cancer. Hence, comprehensive understanding of this pathway might improve development of co-therapy targets for IRE.

## TGF-β Pathway in Pancreatic Cancer and IRE

The transforming growth factor-β (TGF-β) pathway mediates diverse cellular processes and is a major contributor to cancer initiation and progression. In pancreatic cancer, TGF-β plays a paradoxical role as both a tumor suppressor and a tumor promoter. TGF-β family members are upregulated in pancreatic cancer, and increased expression of type II TGF-β receptor is linked with reduced survival in pancreatic cancer patients (71). Intriguingly, in the rabbit VX-2 breast cancer model, it was found that IRE improved the antitumor immune response by lowering the plasma levels of soluble interleukin-2 receptor (sIL-2R) and TGF-β1 (43). While TGF-β signaling was upstream of many crucial signaling pathways in pancreatic cancer, no significant impact of IRE treatment on TGF-β signaling was found in microarray analysis (23). However, microarray analysis was performed only on samples collected 24 hours post-IRE treatment, but the TGF-β signaling dynamics are dependent on many factors and require comprehensive investigation (72).

TGF-β signaling is important in pancreatic cancer and has been actively or passively implicated after IRE treatment but requires further investigation to fully interpret the immunosuppressive role of TGF-β. Common Treg and MDSC cells are subsets of suppressor cells known to have a role in tumor immunology. Treg1 (CD4+CD25−DX5+LAG-3+FoxP3−) is a subset of T regs and have been found to secrete high amounts of IL-10 and TGF-β. TGF-β and IL-10 expression decrease synthesis of IFNγ (**Figure 2**) and TNF-α by pro-inflammatory CD4 T cells, and in turn reduce tumor-specific cytotoxicity of CTLs, prevent activities of dendritic cells and NK cells, and result in tolerance to tumor cells (73–75). Reduction of Tregs after IRE treatment in LAPC patients has been observed (44). Previous studies indicated that MDSC mediate development of Treg cells in a TGF-β-dependent manner (76, 77). MDSCs remained the same on day 2 post-treatment with Nano-Pulse stimulation but significantly decreased on day 7 in LAPC patients (78). In agreement with the reduction of Tregs, IRE along with PD1 blockade and TLR7 agonist decreased MDSC levels on day 7 (21). This indicates the importance of elucidating underlying mechanism involving TGF-β, Tregs and MDSCs.

## TLR3/TLR9 Pathway in Pancreatic Cancer and IRE

Toll like receptor signaling recognize a wide variety of pathogen associated molecular patterns (PAMPs) and activate subsequent immune signaling. CpG and IMO-2125 are TLR9 agonists. CpG motifs are considered PAMPs and can act as an agonist of TLR9 when unmethylated. When CpG binds to TLR9, it triggers a conformational shift in the receptor causing MyD88 recruitment and activation of signaling pathways downstream, culminating in NF-κB activation (79) to initiate a cascade of innate and adaptive immune responses (**Figure 2**). TLR9 agonists (CpG, IMO-2125) activate plasmacytoid dendritic cells to secrete type I interferon and to express increased levels of co-stimulatory molecules such as CD80 and CD86. This is thought to induce a variety of secondary effects, including secretion of cytokines/chemokines, activation of natural killer (NK) cells, and expansion of T-cell populations (80, 81). A humoral immune response is also initiated as TLR9 agonists enhance differentiation of B cells into antibody-secreting plasma cells, potentially promoting antibody-dependent cellular cytotoxicity (82).

In a mouse lymphoma model, IRE along with TLR3/9 agonist and PD1 blockade produced strong antigen specific CD8+ T cells and reduced the number of exhausted intratumoral CTLs, resulting in complete removal of primary tumors and distant tumors. IRE combination therapy efficiently altered the tumor microenvironment to promote anti-tumor signaling, as shown by decreased M2 macrophages, MDSCs, plasmacytoid dendritic cells, and Tregs and by increased M1 macrophages and CTLs (83). A phase II clinical trial PANFIRE-II (NCT01939665) showed that IRE resulted in a median overall survival of 17 months after diagnosis when combined with 5-fluorouracil, leucovorin, irinotecan, and oxaliplatin (84). PANFIRE-III trial (NCT04612530) is at present assessing safety and efficacy of IRE + systemic anti-PD-1 ± intratumoral TLR-9 agonist in metastatic PDAC patients (85). While clinical trials are underway it should still be noted that pattern recognition receptor pathways have not been well studied in the context of IRE and pancreatic cancer.

## Conclusion

IRE is a promising and novel method to treat pancreatic cancer. Many important biochemical pathways have been implicated in pancreatic cancer and several of them were found altered after IRE treatment using human pancreatic cancer patients or animal models. For many of those pathways, starting and endpoints have been tested after IRE treatment but comprehensive knowledge of the alterations of the components of a whole pathway is crucial to understand and design an effective target for treatment. A timeline of initial tumor growth, tumor reduction after IRE treatment, IFNγ expression and its stimulation of PD-1/PD-L1, activation/suppression of pattern recognition receptors, and the TGF-β pathway would help the field develop co-therapy targets and design improved clinical trials.

## Author Contributions

KI wrote the first draft of the manuscript. KI, MN-S, and RB contributed to the design of the figures. All authors reviewed and commented on the subsequent drafts of the manuscript. All authors contributed substantially to the article and approved the final version.

## Funding

This work was supported, in part, by the Virginia Maryland College of Veterinary Medicine, The Virginia Tech Institute for Critical Technology and Applied Sciences Center for Engineered Health, and The National Institutes of Health R21EB028429 (ICA) R01CA213423 (RVD). The content is solely the responsibility of the authors and does not necessarily represent the official views of the NIH or any other funding agency.

## Conflict of Interest

Authors IA, NA, and RD have patent applications on this work.

The remaining authors declare that the research was conducted in the absence of any commercial or financial relationships that could be construed as a potential conflict of interest.

## Publisher’s Note

All claims expressed in this article are solely those of the authors and do not necessarily represent those of their affiliated organizations, or those of the publisher, the editors and the reviewers. Any product that may be evaluated in this article, or claim that may be made by its manufacturer, is not guaranteed or endorsed by the publisher.
